# Psychiatric comorbidities among patients hospitalized in the addictology department of Ar Razi hospital in Morocco

**DOI:** 10.1192/j.eurpsy.2024.848

**Published:** 2024-08-27

**Authors:** Y. Bensalah, M. Sabir, F. Elomari

**Affiliations:** psychiatric hospital Ar Razi, Salé, Morocco

## Abstract

**Introduction:**

Comorbidity between psychiatric disorders and disorders linked to psychoactive substance use is common and represent a real public health problem.

The association of a psychiatric disorder can, in certain cases, modify the treatment methods and also the evolution of the addictive behavior.

**Objectives:**

Determining the prevalence of psychiatric comorbidities in patients with substance use disorder Identify the sociodemographic and clinical characteristics of patients hospitalized in the addictology department.

**Methods:**

We conducted a cross-sectional study with descriptive and analytical aims, in order to study psychiatric comorbidities in 150 patients with substance use disorder hospitalized in the addictology department of Ar Razi hospital in Salé over a period from June 1, 2022 to August 30, 2023.

Data collection was done using a questionnaire including clinical and socio-demographic characteristics, the prevalence of problematic use of psychoactive substances and the comorbidity of psychiatric disorders (diagnoses assessed by DSM 5 criteria).

**Results:**

A male predominance was noted (80%). The main substances consumed in the last 12 months were tobacco (98%), cannabis (74%), alcohol and benzodiazepines.

The majority of patients presented at least one psychiatric comorbidity (80%), with a predominance of depressive disorder and anxiety disorders.

Personal history of suicide attempts was found in 30% of the sample Substance dependency that prompted initially the consultation was higher in patients with psychiatric comorbidity (p < 0.05)

Post-traumatic stress disorder was significantly associated with the presence of problematic cocaine and alcohol use. Social phobia is associated with the absence of a criminal record.

**Conclusions:**

Addictive behaviors are often associated with psychiatric disorders. The most common psychiatric comorbidities are depression, anxiety and personality disorders, hence the need for simultaneous treatment of psychiatric pathologies and addictive behavior

**Disclosure of Interest:**

None Declared

